# Oriental Sore

**Published:** 1912-09

**Authors:** 


Oriental Sore.—S. T. Darling and A. B. Herrick, *Proceedings of the Canal Zone Med. Assn.*, Oct., 1910—March, 1911, report cases. The first was autochthonous. A protozoan closely resembling the Leischmannia Tropica was found and infection by flies, etc., seems highly probably.

